# A cold-responsive *fimACD* chaperone–usher operon tunes motility and biofilm formation in *Pseudomonas fragi* D12

**DOI:** 10.1128/aem.02472-25

**Published:** 2026-04-20

**Authors:** Shaoyu Li, Xia Zhang, Murong Li, Meng Zhou, Zhihao Xing, Wenbo Zhu, Yehui Liu, Qiyun Li, Xueli Zang, Sitong Zhang

**Affiliations:** 1 The Key Lab of Straw Comprehensive Utilization and Black Soil Protection, Ministry of Education, Jilin Agricultural University 85112, Changchun, People's Republic of China; 2 College of Life Sciences, Jilin Agricultural University 85112, Changchun, People's Republic of China; 3 Jilin Agricultural Environmental Protection and Rural Energy Management Station, Changchun, People's Republic of China; 4 Changchun Medical College 382027 , Changchun, People's Republic of China; University of Milano-Bicocca, Milan, Italy

**Keywords:** *Pseudomonas fragi*, fim operon, motility, cold adaptation, c-di-GMP signaling

## Abstract

Low temperature alters bacterial growth and surface-linked behaviors; however, the genetic role of the associated pilus systems in cold adaptation remains unclear. Here, we used the psychrotolerant tundra isolate *Pseudomonas fragi* D12 as a model to investigate the transcriptional responses and functional divergence of three fimbrial genes, *fimA*, *fimC*, and *fimD,* through a combination of transcriptome analysis and gene knockout/overexpression assays. RNA-seq analysis revealed that extreme cold stress (4°C) triggered a robust induction of the fim cluster and an adjacent regulatory module comprising an Arc-family DNA-binding protein and an EAL-domain phosphodiesterase. qRT-PCR confirmed the RNA-seq trends. Functional assays demonstrated distinct ecological roles; deletion of *fimA* increased swimming but reduced swarming, whereas overexpression of *fimA* led to an increase in swarming. *fimC* overexpression enhanced swimming, whereas *fimC* deletion decreased swarming. *fimD* deletion increased swimming and reduced swarming, while *fimD* overexpression suppressed swarming. Temperature-gradient experiments further showed that across the three temperatures examined (4°C, 15°C, and 30°C), motility and biofilm formation were the highest at 15°C. Transmission electron microscopy associated these behavioral changes with altered fimbrial density and organization, and growth-curve analysis indicated no major defects in planktonic proliferation. In combination, the data point to a fimbrial apparatus that is transcriptionally responsive to cold and may mechanically modulate the coupling of a single polar flagellum to liquid and solid interfaces, while the genomic context of *fimACD* remains compatible with local modulation of cyclic di-GMP signaling that has yet to be examined directly.

**IMPORTANCE:**

Low-temperature environments are widespread in nature; however, the genetic contributions of bacterial surface appendages to cold-associated behavioral adaptation remain poorly understood. Our work, using the psychrotolerant tundra isolate *Pseudomonas fragi* D12, offers a tractable example in which a single chaperone–usher fimbrial operon exerts a marked influence on how cells move and form biofilms across the three temperatures examined (4°C, 15°C, and 30°C). By combining transcriptomics, defined genetic changes, and imaging, we connect cold-inducible expression of the *fimACD* locus with altered fimbrial architecture, motility behavior, and biofilm robustness, while separating these effects from bulk growth. The results support a view in which fimbriae in a psychrotolerant bacterium operate as adjustable elements that influence when cells favor long-range swimming versus surface-associated growth. Such information may provide direct genetic and phenotypic evidence for functional specialization of the fimbrial system under cold stress, offering new insight into the molecular strategies that enable microbial survival in low-temperature habitats.

## INTRODUCTION

Low-temperature environments are widespread across the Earth’s surface, encompassing polar permafrost, deep-sea sediments, alpine glaciers, and artificial cold-storage systems. These niches constitute both long-term habitats and intense physiological stressors for microorganisms. Cooling reduces membrane fluidity, slows enzyme-catalyzed reaction rates, and impairs protein-folding efficiency, precipitating systemic adjustments in metabolism and community composition (1, 2). Microorganisms that adapt to such conditions often deploy a suite of biochemical and structural strategies, including the modulation of membrane lipid unsaturation, rapid accumulation of cold-shock proteins and molecular chaperones, active uptake or synthesis of compatible solutes, and shifts toward a communal (sessile) lifestyle (3–5). These mechanisms not only ensure individual survival but also shape biogeochemical cycling and energy flux in cold ecosystems.

Against this background, regulation of bacterial surface structures has attracted growing attention. The trade-off between motility and attachment is viewed as a key ecological strategy that determines whether microorganisms can establish long-term colonization in cold environments (6). In many gram-negative bacteria, the second messenger cyclic di-GMP (c-di-GMP) network affects a reversible transition from swimming to surface attachment by coordinating flagellar synthesis, exopolysaccharide production, and pilus assembly (7, 8). Thermal cues can modulate this network via thermosensitive diguanylate cyclases or temperature-dependent transcriptional modules, thereby triggering behavioral switches across different temperature ranges (9). Maintaining active swimming at higher temperatures while switching to surface attachment and biofilm formation at low temperatures thus appears to be a widespread strategy among cold-adapted bacteria.

Pili assembled by the chaperone–usher (CU) pathway are common adhesins in gram-negative bacteria, and type-1 (I-type) pili are among the best-characterized examples (10, 11). This system is generally encoded by a fim operon, where *fimA* codes for the primary pilin subunit (12); *fimC* encodes a periplasmic chaperone that not only aids in proper subunit folding but also prevents premature aggregation in the periplasmic space; and *fimD* is responsible for encoding an outer-membrane usher, which plays a key role in assembling the pilus by providing the platform for polymerization and facilitating its translocation to the cell surface (13–15). In pathogenic strains, the *fim* genes are tightly linked to how the bacteria attach to the host and dodge the immune system (16). However, systematic genetic investigations of *fim*-type pili in non-pathogenic bacteria, particularly under low-temperature conditions, remain scarce; existing reports are largely restricted to ambient temperatures and pathogenic models (17).

Earlier work (18) used *P. fragi* D12 to find genes related to cold adaptation, but that study did not explore how surface structures contribute to the bacteria’s response to cold. In our study, we combined temperature-gradient transcriptomic profiling with targeted gene manipulation (deleting and overexpressing *fimA*, *fimC*, and *fimD*) to gain a deeper understanding of how *fim* operon genes behave in *P. fragi* when faced with cold stress. By connecting gene expression changes to the bacteria’s behavior and cell structure, we not only reveal a specific ecological role for *fim*-type pili in cold-adapted *Pseudomonas* but also provide new theoretical insight and potential targets for understanding behavioral regulation in low-temperature microbial communities.

## RESULTS

### Genome response to cold stress and structural analysis of the *fim* operon

**Fig 1:**
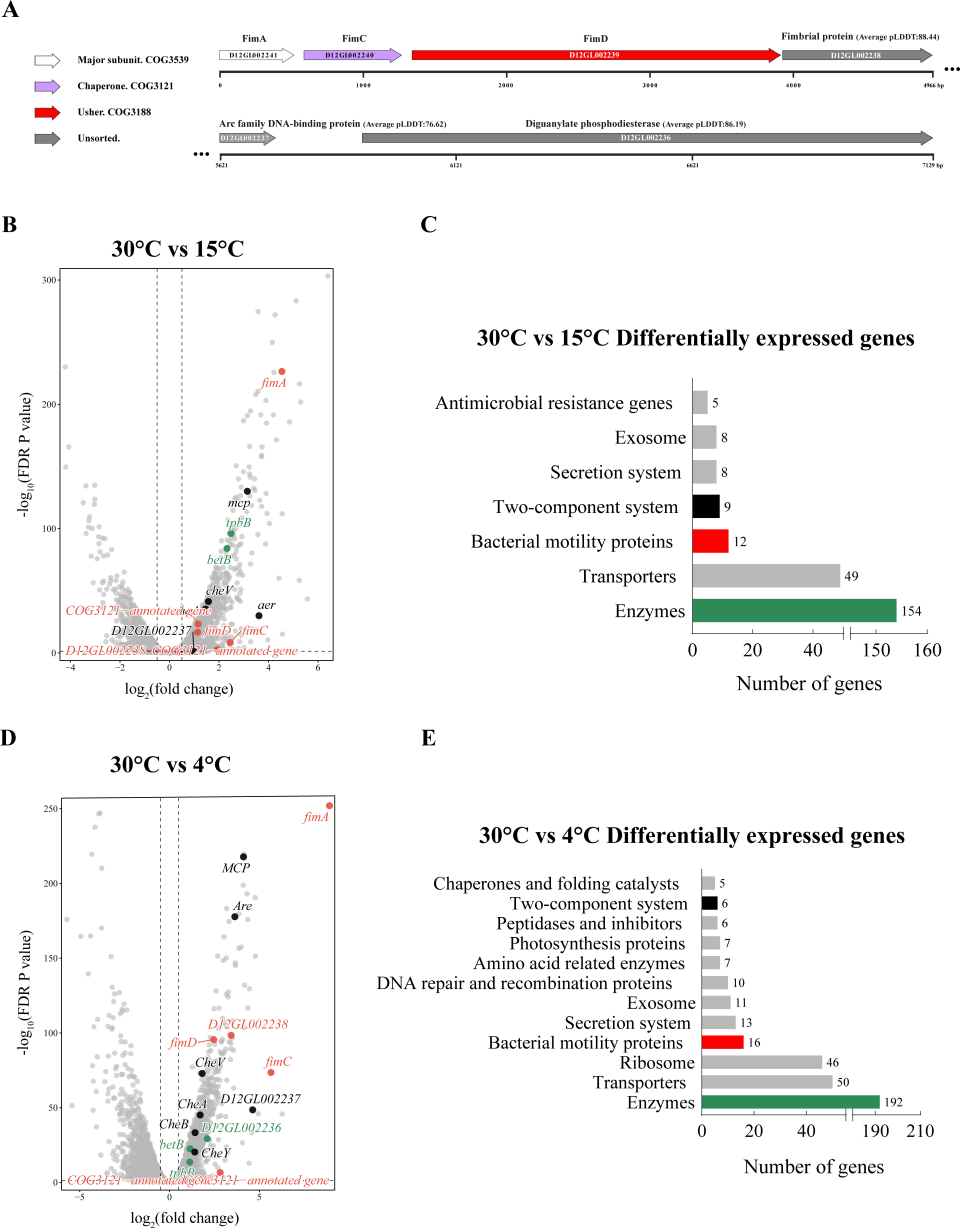
Genomic organization and temperature-dependent transcriptional profiling of the *fim* locus in strain *P. fragi* D12. (**A**) Schematic representation of the *fim* operon and downstream regulatory module (NCBI Accession no. CP104861). The core cluster comprises the major subunit (fimA), chaperone (fimC), and usher (fimD), followed by an unannotated fimbrial protein (D12GL002238). A downstream functional module includes an Arc family DNA-binding protein (D12GL002237) and a diguanylate phosphodiesterase (D12GL002236). Average pLDDT scores from AlphaFold structural predictions are indicated for unannotated and downstream genes. (**B and D**) Volcano plots illustrating differentially expressed genes (DEGs) between 30°C and 15°C (**B**) and between 30°C and 4°C (**D**). DEGs were defined as |log_2_FC| > 1 and *P* < 0.05. At 15°C, *fimA* and chemotaxis regulators were induced, while downstream components (D12GL002237 and D12GL002238) showed negligible changes. At 4°C, a stronger global response was observed, with significant upregulation of the entire *fim* cluster, the Arc family protein, and the diguanylate phosphodiesterase. (**C and E**) Functional classification of DEGs at 15°C (**C**) and 4°C (**E**) based on KEGG enrichment and category analysis. The x-axis represents the number of genes associated with each functional group. The predominant enrichment in "bacterial motility proteins" and "two-component system" suggests a coordinated shift in environmental adaptation strategies under cold stress. Color coding is consistent with the volcano plots.

To elucidate the genetic basis of surface attachment in *P. fragi* D12, we first characterized the structural components of the *fim* locus. Operon gene mapping revealed a core cluster consisting of the major subunit (fimA), a chaperone (fimC), and an usher protein (fimD) (Fig. 1A). Notably, the classical regulatory elements fimB and fimE, which typically mediate site-specific recombinational switching in enteric bacteria, were absent from this locus.

Immediately downstream of *fimD*, we identified an additional, previously unannotated fimbrial protein (D12GL002238). To assess the structural integrity of this and other unsorted proteins within the locus, 3D structure modeling was performed using the AlphaFold Protein Structure Database. The unannotated fimbrial protein exhibited a high average predicted local distance difference test (pLDDT) score of 88.44, suggesting a highly confident structural fold. Furthermore, the downstream region of the operon contains a distinct putative module comprising an Arc family DNA-binding protein (pLDDT: 76.62) and a protein reminiscent of a diguanylate phosphodiesterase (pLDDT: 86.19), the latter potentially involved in the degradation of the secondary messenger c-di-GMP (Fig. 1A).

Temperature is a major determinant of microbial adaptation, and under cold conditions, bacteria commonly reprogram gene expression to mount stress responses (19). To investigate this, the strain was cultured at 30°C (control) and 15°C (moderate cold stress), and transcriptomes were compared. A total of 261 differentially expressed genes (DEGs) were identified (|log_2_ fold change| > 1, FDR-adjusted *P* < 0.05; Fig. 1B). While several genes involved in osmotic regulation (*betB*, |log_2_FC| = 2.3) and c-di-GMP synthesis (*tpbB*, |log2FC| = 2.4) were significantly upregulated, the downstream components of the *fim* operon remained largely unchanged. Specifically, the Arc-family protein (D12GL002237) and the unannotated fimbrial protein (D12GL002238) showed negligible induction (|log_2_FC| of 0.98 and 0.35, respectively), while the phosphodiesterase (D12GL002236) was not significantly detected as a DEG at this temperature.

When cultures were shifted to extreme cold stress (4°C), the number of DEGs rose markedly to 361 (Fig. 1D). Under these conditions, the entire *fim* cluster and its downstream regulatory module were strongly induced. The major pilus structural gene *fimA* showed a dramatic upregulation (|log_2_FC| = 8.9). Parallel to this, the Arc family DNA-binding protein (D12GL002237) and the unannotated fimbrial protein (D12GL002238) were significantly upregulated with |log_2_FC| values of 4.63 and 3.43, respectively. Furthermore, the diguanylate phosphodiesterase (D12GL002236) was also induced (|log_2_FC| = 2.10). This coordinated upregulation at 4°C, but not at 15°C, suggests a specialized response to extreme cold, where the strain may enhance attachment and colonization through fimbrial production while simultaneously modulating c-di-GMP signaling to fine-tune its environmental adaptation.

Further KEGG enrichment analysis (Fig. 1C and E) showed that DEGs at 4°C were predominantly associated with “two-component system” and “bacterial motility proteins,” indicating that extreme temperature shifts trigger a comprehensive reconfiguration of stress perception and extracellular behavior in strain *P. fragi* D12.

To validate the RNA-seq results, 20 DEGs showing high expression and large inter-sample fold changes were randomly selected for qRT-PCR (three biological replicates per group), with 16S rDNA used as the reference gene. Expression changes are presented as log_2_ (fold change), and Pearson correlation was used to compare qRT-PCR fold changes with RNA-seq results (Fig. S1). The two methods showed a strong linear correlation: r = 0.974 and 0.993 (both *P* < 0.001); the linear regression models yielded R² = 0.949 and 0.985, respectively, supporting the reliability of the RNA-seq–derived DEGs.

### Effects of pilus genes on motility at low temperature and pilus architecture

**Fig 2:**
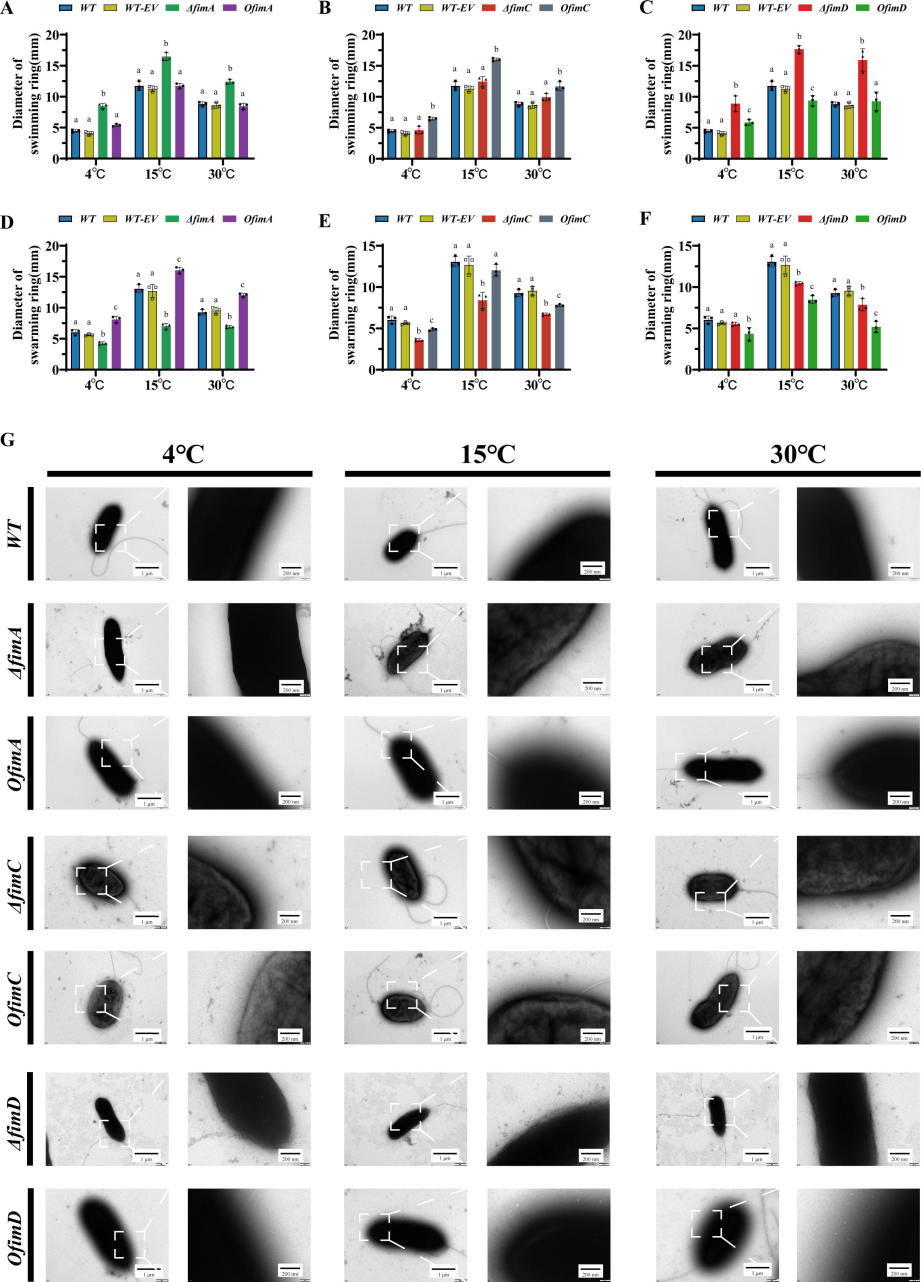
Regulatory effects of *fimA*, *fimC,* and *fimD* on *P. fragi* D12 motility and surface morphology across temperatures. (**A–C**) Swimming ability of wild type (WT), wild type harboring the empty vector (WT-EV), gene deletion strains (Δ*fimA*, Δ*fimC*, and Δ*fimD*), and overexpression strains (*OfimA*, *OfimC*, and *OfimD*) under three temperature conditions (4°C, 15°C, and 30°C). Bar graphs show swim-zone diameters; data are mean ± SD of three independent biological replicates (*n* = 3). Statistical significance was assessed by one-way ANOVA, followed by Tukey’s multiple comparisons; different letters denote statistically significant groups within the same temperature (same letter, *P* ≥ 0.05; different letters, *P* < 0.05). (**D–F**) Corresponding results for swarming ability, grouping, temperature conditions, statistical tests, and annotation conventions are the same as in panels A to C. All experimental conditions (medium composition, inoculation method, incubation time, and measurement procedures) are described in Materials and Methods. (**G**) Negative-stain TEM images showing surface ultrastructure and fimbrial features for the three strain classes at the indicated temperatures. Each main panel includes a local magnification (lower right, corresponding to the white boxed area). Scale bars: main panels = 1 µm; insets = 200 nm. Images are representative fields; sample preparation and microscope model are detailed in Materials and Methods.

Across 4°C, 15°C and 30°C, the overall motility of all strains peaked at 15°C (Fig. 2). Against this common background, manipulation of different fimbrial genes produced highly comparable but directionally divergent effects: the two motility modes, swimming and swarming, exhibited a trade-off under genetic control, and these behavioral differences could be accounted for by changes in fimbrial number and arrangement (20, 21). A wild-type strain carrying the empty vector used for overexpression constructs (WT+EV) was included as a control; its swimming and swarming diameters were indistinguishable from those of the wild-type strain at all temperatures tested (Fig. 2A through F).

Specifically, comparisons under the 15°C condition revealed clear patterns. Deletion of *fimA* increased the swimming zone from 11.7 ± 0.6 mm in the wild type to 16.6 ± 0.6 mm (*P* < 0.01) (Fig. 2A). Conversely, swarming changed in the opposite direction: the wild type measured 13.2 ± 0.6 mm, the *ΔfimA* strain decreased to 6.9 ± 0.4 mm, and *fimA* overexpression increased swarming to 16.0 ± 0.5 mm (Fig. 2D). These data suggest that *fimA* abundance influences the balance between swimming-based dispersal and surface-associated colonization.

By contrast, manipulation of *fimC* predominantly promoted swimming. At 15°C, *fimC* overexpression raised the swimming zone to 16.0 ± 0.2 mm, significantly greater than the wild type (11.7 ± 0.6 mm; *P* < 0.01) (Fig. 2B). The effect on swarming was comparatively attenuated, with no significant enhancement observed (Fig. 2E). This indicates that elevated *fimC* levels correlate with a shift toward a planktonic lifestyle, likely by reducing the density of adhesive surface structures.

*fimD* displayed a markedly negative regulatory profile: deletion of *fimD* enhanced swimming at all temperatures, with a swimming zone of 17.6 ± 0.6 mm at 15°C (vs. 11.7 ± 0.6 mm for the wild type; *P* < 0.001), whereas *fimD* overexpression suppressed swimming to 9.3 ± 0.8 mm (Fig. 2C). In swarming assays, the *ΔfimD* strain was reduced relative to the wild type but remained higher than the overexpression strain (Fig. 2F). Overall, *fimA* and *fimC* acted in opposition, while *fimD* exerted a strong negative influence on both motility modes.

Transmission electron microscopy (TEM; Fig. 2G) provided structural corroboration for these phenotypes: *fimA* overexpression produced a marked increase in fimbrial density with a compact arrangement, whereas *ΔfimA* showed fewer fimbriae but retained characteristic filaments; *ΔfimD* lacked fimbriae, displaying a smooth surface, while *fimD* overexpression yielded dense, disordered fimbrial arrays; and *fimC* overexpression was associated with a reduction in fimbrial number. These observations indicate that changes in gene expression modulate fimbrial number and arrangement, which, at the ultrastructural level, drive the behavioral allocation between swimming and swarming. In addition to pili, TEM inspection did not reveal the loss of polar flagella or obvious shortening of flagellar filaments in any deletion or overexpression strain.

qRT-PCR analysis verified the intended genetic manipulations of the fim operon genes (Fig. S2). Transcripts of *fimA*, *fimC*, and *fimD* were nearly undetectable in the corresponding deletion strains and increased several-fold in the respective overexpression strains. In contrast, transcript levels of the flagellar regulator and structural genes *fleQ*, *fliA*, *flgB*, *flgE*, and *fliC* did not differ significantly among the wild type, WT+EV, deletion strains, and overexpression strains (Fig. S3). Together with the TEM observations, these data indicate that the motility changes described above arise in the context of intact polar flagella and are associated with altered fimbrial architecture rather than gross changes in flagellar gene expression or structure.

### Pilus genes and biofilm formation

**Fig 3:**
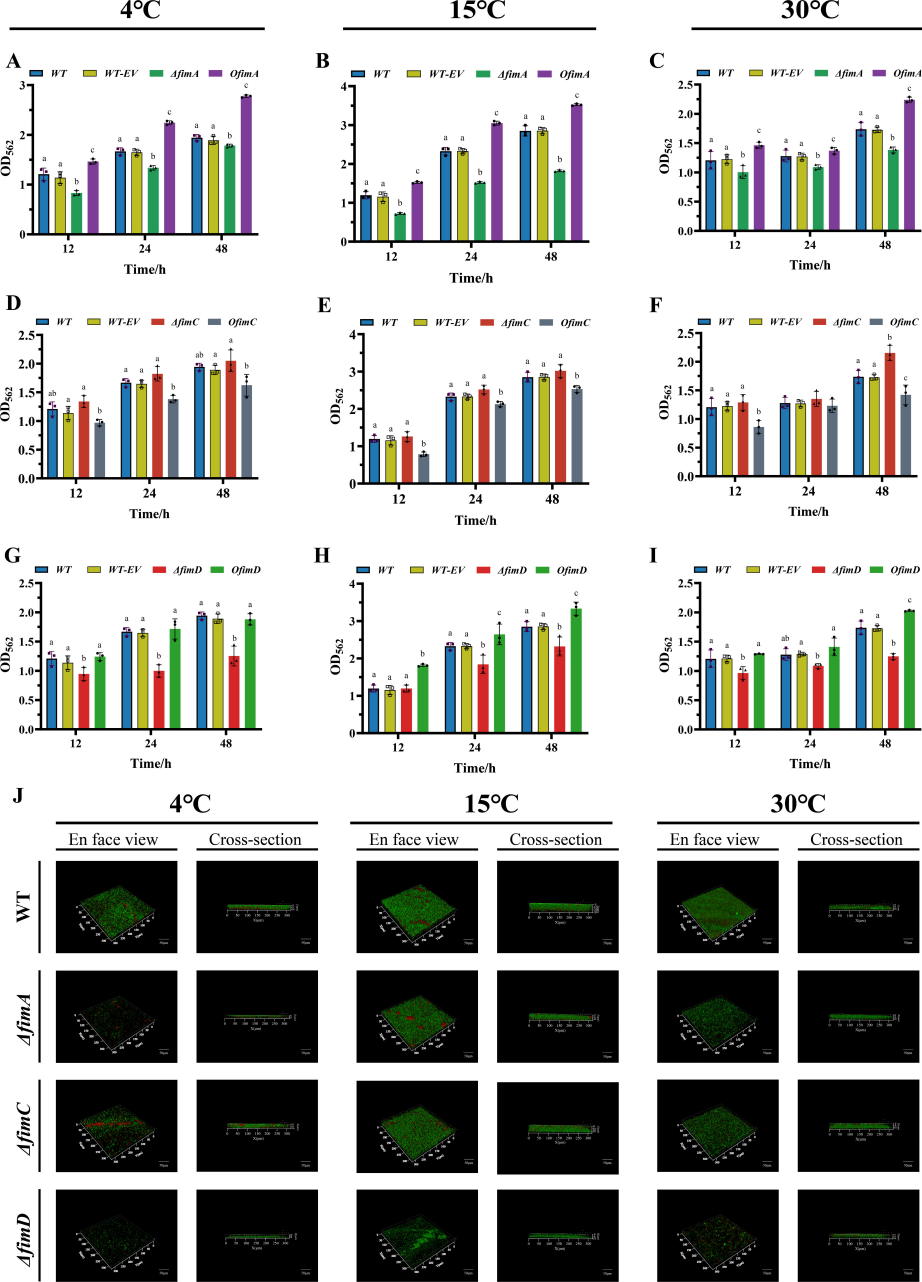
Effects of *fimA*, *fimC,* and *fimD* on *P. fragi* D12 biofilm formation at different temperatures. (**A–I**) Biofilm biomass was quantified by crystal-violet staining of the indicated genotypes at 12, 24, and 48 h under each temperature. Comparisons are among wild type (WT), wild type harboring the empty vector (WT-EV), deletion mutants (Δ*fimA*, Δ*fimC*, and Δ*fimD*), and overexpression strains (*OfimA*, *OfimC*, and *OfimD*). The results are presented as OD_590_ and are shown as mean ± SD from three independent biological replicates (*n* = 3). Statistical analysis was performed by one-way ANOVA, followed by Tukey’s multiple-comparisons test; letters indicate differences at the same time point. Identical letters denote no significant difference (*P* ≥ 0.05), and different letters denote significant differences (*P* < 0.05). (**J**) Morphological characterization of biofilms at 48 h. Top row: top views; bottom row: side views. Representative fields from three independent experiments are shown. Scale bars are indicated on the images.

To systematically evaluate the contribution of fimbrial genes to biofilm formation at different temperatures, we quantitatively and microscopically assessed the biofilm-forming capacity of deletion mutants, overexpression strains, and the wild-type for *fimA*, *fimC,* and *fimD* at 4°C, 15°C, and 30°C (Fig. 3). The WT+EV control formed biofilms with biomass and architecture comparable to the wild type under all tested conditions, indicating that the overexpression vector and associated antibiotics alone did not alter biofilm formation. Overall, all strains exhibited the most active biofilm formation at 15°C, significantly greater than at 4°C and 30°C, consistent with previous reports that moderate temperatures promote collective microbial behaviors (22).

At the genotypic level, *fimA* and *fimD* displayed concordant positive regulatory effects. At all three temperatures, overexpression strains produced thicker biofilms, whereas the corresponding deletion mutants showed the opposite phenotype. For example, at 15°C and 24 h, the crystal-violet absorbance of the *fimA* overexpression strain reached 3.05 ± 0.50 *A*_562_, which was significantly higher than that of the wild type (WT: 2.30 ± 0.90 *A*_562_; *P* < 0.001), while *ΔfimA* measured 1.52 ± 0.30 *A*_562_ (*P* < 0.001). This divergence widened at 48 h (*OfimA* = 3.52 ± 0.30 *A*_562_ vs. WT = 2.87 ± 0.13 *A*_562_; *ΔfimA* = 1.82 ± 0.30 *A*_562_; *P* < 0.001) (Fig. 3B). *fimD* exhibited an almost identical pattern; at 15°C, the biofilm biomass of *fimD* overexpression at 48 h reached 3.31 ± 0.17 *A*_562_, whereas *ΔfimD* was 2.37 ± 0.24 *A*_562_, a significant decrease relative to WT (2.87 ± 0.13 *A*_562_; *P* < 0.05) (Fig. 3H). Collectively, these results indicate that *fimA* and *fimD* exert a sustained promotive effect on biofilm formation.

By contrast, *fimC* acted as a negative regulator (Fig. 3D through F). *fimC* overexpression exhibited weaker biofilm formation than WT under most tested conditions, while *ΔfimC* generally showed no significant reduction. At 15°C and 48 h, the absorbance of *fimC* overexpression was 2.51 ± 0.09, significantly lower than WT (2.87 ± 0.13 *A*_562_; *P* < 0.05), whereas *ΔfimC* measured 3.01 ± 0.18 *A*_562_ (*P* > 0.05) (Fig. 3E). Similar directional trends were observed at 4°C and 30°C; although the absolute biomass was lower, the genotype-dependent differences remained consistent.

This pattern is supported by both quantitative measurements and morphological observations. Confocal microscopy (Fig. 3J) revealed that at 15°C, biofilms formed by *ΔfimA* and *ΔfimD* were visibly sparser and reduced in thickness, whereas *ΔfimC* retained a thickness comparable to WT. At 4°C and 30°C, biofilms were generally thin. qRT-PCR analysis of the *fim* operon confirmed that transcript levels of downstream genes were not detectably altered in the deletion and overexpression strains under the conditions tested (Fig. S1), arguing against polarity effects as an explanation for the biofilm phenotypes. Together, the quantitative and microscopic evidence validate the modulatory roles of *fimA*, *fimC,* and *fimD* in strain biofilm formation.

### Effect of pilus genes on strain growth at three temperatures

**Fig 4:**
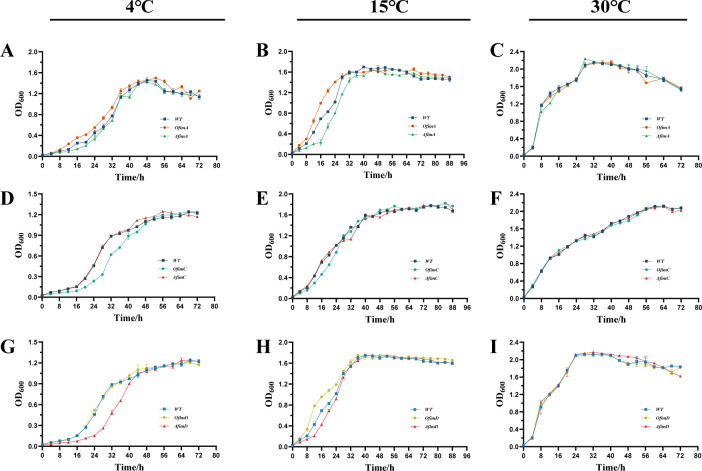
*In vitro* growth curves OD_600_ of *P. fragi* D12 with different genotypes. Comparisons are among wild type (WT), wild type harboring the empty vector (WT-EV), deletion mutants (Δ*fimA*, Δ*fimC*, and Δ*fimD*), and overexpression strains (*OfimA*, *OfimC*, and *OfimD*). All strains were inoculated at a 1% (vol/vol) ratio into Luria–Bertani (LB) and incubated at 4°C (**A, D, G**), 15°C (**B, E, H**), and 30°C (**C, F, I**). Growth was monitored by measuring the optical density at 600 nm (OD_600_) every 4 h; monitoring continued to 72 h at 4°C and to 88 h at 15°C and 30°C. Although transient differences in lag phase or exponential growth rates were observed at 4°C and 15°C, stationary-phase biomass remained comparable across all genotypes, indicating that fimbrial gene manipulation does not significantly impair overall planktonic fitness.

To distinguish the specific effects of fimbrial genes on biofilm formation from general constraints on growth, OD_600_ was recorded every 4 h for the wild-type (WT) strain and for *fimA*, *fimC*, and *fimD* deletion and overexpression strains at 4°C, 15°C, and 30°C, and the corresponding growth curves were plotted (Fig. 4). Across the different incubation conditions, the growth trajectories of all strains were broadly similar to those of the WT; some fluctuations occurred during the exponential phase, but stationary-phase OD_600_ values were comparable. At 30°C, the growth curves of the WT, deletion mutants, and overexpression strains nearly coincided. All cultures entered the exponential phase within approximately 2–3 h and reached the stationary phase at about 12 h, with similar maximal OD_600_ values (e.g., WT = 2.10 ± 0.05, *OfimA* = 2.15 ± 0.08; *P* > 0.05; Fig. 4C, F, and I). Within the resolution of OD_600_ measurements, which assess turbidity as a proxy for biomass rather than exact cell counts, plasmid maintenance and antibiotic supplementation before inoculation did not impose a detectable fitness cost under these optimal conditions.

At 15°C, growth of all genotypes was slower, and nearly all strains entered the exponential phase 4–6 h after inoculation. During exponential growth, *fimA* and *fimD* overexpression strains showed higher OD_600_ values than the WT at intermediate time points; for example, at 20 h, the mean OD_600_ of *fimA*/*fimD* overexpression strains was ~1.23 ± 0.02, whereas the WT reached only ~0.83 ± 0.09 (*P* < 0.05; Fig. 4B and H). In contrast, deletion of *fimA* or *fimD* reduced growth rates between 12 and 24 h, but OD_600_ differences had largely disappeared by 48 h (e.g., WT = 1.67 ±0 .06, *ΔfimA* = 1.63 ± 003; *P* > 0.05). This pattern indicated that at 15°C, the deletion and overexpression mutants exhibited only transient delays or accelerations in growth, rather than persistent defects in the final culture yield. Consistent with this, the pronounced increases or decreases in biofilm formation observed for these strains at 15°C (Fig. 3B and H) were unlikely to be explained by large differences in planktonic growth capacity.

At 4°C, growth of all strains was markedly reduced, as expected for cultivation near the lower boundary of the permissive temperature range. Under these conditions, the *ΔfimA* and *ΔfimD* mutants displayed prolonged lag phases: the WT entered exponential growth at ~12 h, whereas *ΔfimD* did not do so until ~20 h. This delay was temporary; both deletion strains resumed growth and reached OD_600_ values at 48 h that were close to that of the WT (e.g., *ΔfimD* = 1.08 ± .03, WT = 1.10 ± .03; *P* > 0.05; Fig. 4A and G). Final cell densities were therefore sufficient to support biofilm formation, and the reduced biofilm biomass of *ΔfimA* and *ΔfimD* is more likely attributable to defects in adhesive structures. Previous studies have noted that the lag phase is a highly dynamic and evolvable stage of adaptation, in which many genetic changes modify growth kinetics only during lag or early exponential phase without necessarily altering stationary-phase cell density (23).

On this basis, analysis of the growth curves indicated that *fimA*, *fimC*, and *fimD* regulated biofilm formation mainly by influencing surface attachment, rather than by imposing major limits on planktonic proliferation within the temperature range tested. Although subtle growth effects below the detection threshold of OD_600_ measurements cannot be completely excluded, the data do not support changes in overall growth as the primary cause of the observed biofilm phenotypes.

## DISCUSSION

### Hypothetical role of a local c-di-GMP signaling module in modulating *fim* function

The regulation of type I fimbriae is classically associated with the fimBE-mediated phase variation switch found in *Enterobacteriaceae*, which controls expression through DNA inversion (24). However, these recombinases were not detected in the bioinformatic analysis of the *P. fragi* D12 genome, potentially indicating that this strain relies on a distinct strategy for fimbrial assembly regulation. This divergence prompted a detailed examination of the chromosomal region flanking the *fimACD* cluster to identify potential regulatory elements that substitute for the canonical phase-variation machinery (Fig. 1A).

Immediately downstream of *fimD*, the gene D12GL002238 encodes a predicted fimbrial protein with a high-confidence AlphaFold model, consistent with a minor subunit or adhesin-like component that occupies the pilus tip and confers receptor specificity, similar to accessory subunits in canonical type 1 pilus systems. Beyond this structural block, the adjacent genes D12GL002237 and D12GL002236 form a putative regulatory module. D12GL002237 is predicted to encode an Arc-family DNA-binding regulator, whereas D12GL002236 encodes a protein with an AlphaFold fold compatible with EAL-type phosphodiesterase that hydrolyzes c-di-GMP (25, 26). Arc-type regulators are central components of redox-responsive two-component systems that adjust metabolism and, in some organisms, modulate biofilm formation under oxygen limitation (27). The synteny of the fimbrial structural module with an Arc-family regulator and a putative phosphodiesterase leads us to hypothesize that the *fim* locus may reside within a local circuit potentially coupling environmental inputs such as redox state and temperature-dependent respiratory activity to surface-associated behavior via c-di-GMP modulation.

In many bacteria, networks of diguanylate cyclases (GGDEF proteins) and phosphodiesterases (EAL proteins) couple environmental inputs to motility, exopolysaccharide production, and biofilm architecture (28, 29), and several studies indicate that this control is often exerted through spatially restricted “local” signaling modules rather than only through global changes in bulk c-di-GMP. A direct link between c-di-GMP and pilus or fimbrial systems has been described for MSHA-type pili in *Aeromonas veronii*, where c-di-GMP binding to pilus-associated ATPases promotes pilus biogenesis and surface attachment (30), and for *Lysobacter enzymogenes*, in which the response regulator *PilR* coordinately controls type IV pili, intracellular c-di-GMP, and antifungal antibiotic production (31). Work in *Pseudomonas aeruginosa* and other model organisms has likewise established pili, flagella, and extracellular matrix synthesis as recurrent output arms of c-di-GMP signaling cascades (32).

Within this broader framework, our data suggest a plausible, but as yet unproven, connection between temperature-responsive fimbrial control and c-di-GMP signaling in this species. In the transcriptome, *fimA* is strongly induced under cold stress relative to 30°C, and a tpbB-like diguanylate cyclase is upregulated at 4°C compared with 30°C (Fig. 1D), implying that temperature shifts impinge both on the adhesion machinery and on at least one c-di-GMP-metabolizing enzyme. Combined with the presence of a predicted regulatory module adjacent to the *fim* operon, this organization is compatible with a scenario in which the *fim* locus is embedded in a local c-di-GMP circuit that modulates the balance between motility and surface attachment. However, we have not yet quantified intracellular c-di-GMP, measured the enzymatic activity of the downstream phosphodiesterase, or demonstrated direct regulatory coupling between these enzymes and the fimbrial apparatus. Any proposed c-di-GMP–based interpretation of the *fimACD* phenotypes, therefore, remains a working hypothesis grounded in genomic context and analogy to other systems rather than a pathway demonstrated in this organism.

Temperature-dependent regulation of c-di-GMP has been documented in several bacteria, where changes in growth temperature alter c-di-GMP levels and thereby shift biofilm formation, motility, and colony morphology (32). In our strain, both swimming motility and biofilm biomass peak at 15°C, with lower values at 4 and 30°C (Fig. 2 and 3), which points to an intermediate temperature window where cells can transition efficiently between motile and sessile states. One possible explanation is that temperature inputs feed into diguanylate cyclase/phosphodiesterase modules that generate local c-di-GMP gradients at the cell poles, where flagellar and fimbrial machineries reside, similar to arrangements described for type IV pilus systems in other bacteria (33, 34). Our current experiments do not resolve whether such a mechanism operates here, and we therefore refrain from designating the *fimACD*-associated module as a bona fide “cold-adaptation” system. Instead, we interpret the temperature-dependent *fimACD* expression and *fim*-dependent biofilm phenotypes as one component of a broader response to suboptimal temperature.

A rigorous test of the proposed model will require direct interrogation of c-di-GMP dynamics and their genetic control in this strain. Quantitative measurements of c-di-GMP at 4°C and 15°C, for example, using fluorescent biosensors or mass spectrometry-based approaches, combined with targeted disruption or overexpression of the *tpbB* homolog and the downstream phosphodiesterase, would allow the assessment of how these enzymes shape c-di-GMP pools, *fimACD* transcription, motility, and biofilm architecture. Such experiments will be essential to determine whether the *fim* locus is indeed wired into a temperature-sensitive c-di-GMP signaling network or whether its regulation by temperature proceeds through an alternative route independent of this second messenger.

### Potential mechanical modulation of motility by the *fim* operon in *P. fragi* D12

The distinct roles of *fimA*, *fimC*, and *fimD* in swimming and swarming point to a strategy where *P. fragi* D12 reallocates motility. This likely occurs not through the global rewiring of the flagellar regulon, but rather through the mechanical modulation of the interaction between the polar flagellum and the environment. qRT-PCR showed that key flagellar regulators and structural genes (including *fleQ, fliA, flgB, flgE,* and *fliC*) were not differentially expressed across the *fim* variants (Fig. S3), and TEM confirmed intact polar flagella in all strains (Fig. 2G), arguing against transcriptional cross-regulation of the flagellar machinery. Instead, the motility phenotypes tracked closely with quantitative and qualitative changes in the chaperone–usher fimbrial apparatus: hyper-dense, compact fimbrial arrays in the *fimA*- and *fimD*-overexpression strains, reduced but detectable fimbriae in Δ*fimA*, and an almost smooth envelope in Δ*fimD* (Fig. 2). These observations are consistent with the view that surface appendages act as mechanical “modules” that couple or decouple flagellar torque to either bulk liquid or solid interfaces, thereby biasing cells toward swimming or swarming states (35, 36).

In liquid, a dense fimbrial coat is expected to increase hydrodynamic drag and introduce additional steric interactions around the rotating flagellum and its bundle, effects that have been shown in model systems where co-expression of fimbriae and flagella alters cell-interface interactions and slows translational motion when adhesins are overabundant (37–39). The strong suppression of swimming in *fimA* and *fimD* overexpression strains, despite unaltered flagellar gene expression, is therefore readily explained as a form of mechanical braking, in which excessive fimbrial density interferes with efficient torque transmission and bundle coherence. Conversely, the enhanced swimming seen in Δ*fimD* and *fimC*-overexpression backgrounds, which exhibit strongly reduced fimbrial coverage, is consistent with the idea that removing protruding surface structures minimizes envelope roughness and frees the flagellum to operate closer to its intrinsic performance limit.

On semi-solid agar, however, the same fimbrial structures that hinder free swimming can become advantageous by increasing surface coupling (40). Swarming motility is known to require not only flagellar propulsion but also a favorable balance of surface friction, wetting, and hydration, as well as multicellular coordination and surfactant activity (41, 42). In *Pseudomonas* and *Salmonella*, flagella-driven swarms must overcome high friction and limited free water at the agar interface, with surface appendages and extracellular polymers acting to attract and retain a thin lubricating fluid layer and to convert rotational torque into lateral displacement (43, 44). The pronounced swarming defect of Δ*fimA*, together with the concomitant enhancement in the *fimA*-overexpression strain, suggests that the *fimA*-based fimbriae in *P. fragi* D12 operate primarily as surface-anchoring and traction elements, analogous to type 1 fimbriae that increase adhesion, friction, and effective wettability on abiotic surfaces in *Escherichia coli* (45). In this framework, Δ*fimA* cells retain motile flagella but lack sufficient micro-scale contact points to initiate and sustain collective spreading, whereas the dense yet organized fimbrial arrays of the *fimA*-overexpression strain provide abundant anchor sites and improved interface coupling, enabling more efficient conversion of flagellar torque into swarm expansion.

The negative regulatory profile of *fimD* further supports a mechanical control model. As the predicted usher of the fimbrial assembly system, *fimD* overexpression yields highly disordered fimbrial bundles that correlate with the strongest suppression of both motility modes, consistent with a scenario in which misassembled or overabundant fimbriae behave as a disordered brush that both shields the cell surface from productive contact with agar and strongly impedes motion in liquid. In contrast, complete loss of *fimD* abolishes fimbrial structures, which maximizes swimming but still permits residual swarming, presumably via other adhesins or matrix components that partially compensate for the traction deficit. Taken together, these data argue that the *fimACD* module in *P. fragi* D12 functions as a biophysical clutch that redistributes a fixed flagellar capacity between dispersal and surface colonization. In the extreme cold niches of polar tundra where *P. fragi* thrives as a resilient psychrotrophic colonizer, tuning fimbrial density and architecture provides a rapid, resource-efficient means to navigate the trade-off between maximizing long-range swimming in meltwater channels and promoting swarm-based colonization and subsequent biofilm development on mineral or organic surfaces.

### FimACD-dependent control of biofilm initiation and architectural robustness

The *fimACD* operon in *P. fragi* D12 also governs how cells establish and stabilize biofilms at low temperature. Overexpression of *fimA* accelerated initial surface colonization and increased biofilm biomass across all tested temperatures (Fig. 3A throguh C), whereas *fimA* deletion produced thinner, less cohesive communities (Fig. 3J). This pattern agrees with work in *E. coli* and other gram-negative bacteria showing that type 1 fimbriae, built from *fimA*-like major subunits, are key initiators of near-surface residence (46, 47), microcolony nucleation, and early three-dimensional organization of biofilms. Imaging and genetic studies have further shown that such fimbriae form dense intercellular networks that both promote aggregation and buffer environmental stresses during the transition from planktonic growth to surface-associated clusters (48).

The usher *fimD* defines a second major control point. In D12, *fimD* deletion abolished the formation of thick (Fig. 3J), mature biofilms even though initial attachment still occurred, consistent with the central role of *PapC/fimD* ushers as outer-membrane assembly platforms for chaperone–subunit complexes in classical CU systems (49). Structural and biochemical work on *PapC/fimD* homologs shows that disruption or misregulation of the usher stalls pilus polymerization and leaves subunits trapped in periplasmic intermediates (12, 50), which explains why Δ*fimD* cells retain flagella and other envelope components yet fail to elaborate extended fimbrial arrays required for biofilm thickening.

Manipulating the chaperone *fimC* yielded a more complex outcome. Removing *fimC* reduced classical fimbrial fibers in TEM, yet promoted spontaneous aggregation and maintained dense (Fig. 2G), compact biofilms, indicating that alternative adhesion modules can compensate when the canonical type 1 fimbrial pathway is compromised. In *Enterobacteriaceae*, the *PapD/fimC* family belongs to the COG3121 group, and many genomes encode multiple *PapD*-like chaperones, some of which service distinct CU operons or accessory fimbrial systems (51). The presence in the D12 transcriptome of several *PapD*- or COG3121-annotated genes independent of *fimC* suggests that redundant chaperone–usher pairs could assemble additional (Fig. 1B and D), as-yet-uncharacterized fimbrial structures that support aggregation when the main *fimC*-dependent route is disrupted. At the same time, Δ*fimC* biofilms likely draw on fimbriae-independent adhesins. In *E. coli*, the autotransporter Agn43 mediates strong homotypic interactions that drive autoaggregation and robust surface colonization, and recent work shows that *fimC* can bind the *agn43* promoter and modulate *Agn43*-dependent autoaggregation (52). A similar division of labor between CU fimbriae and one or more autotransporter-like adhesins would account for the compact, fimbriae-poor biofilm architecture observed for Δ*fimC* in D12.

In contrast, *fimC* overproduction reduced visible fimbriae and increased swimming motility (16.0 ± 0.2 mm), although polar flagella remained intact. Overexpression of specific periplasmic chaperones is known to generate non-productive complexes and activate envelope quality-control systems; in *E. coli*, the *Cpx* two-component pathway senses misfolded periplasmic pilus subunits, adjusts expression of P pili, and broadly modulates envelope-localized organelles, including type IV pili (53, 54). By analogy, excess *fimC* in D12 is likely to saturate chaperone–subunit binding equilibria and trigger envelope stress responses and proteolytic pathways, thereby lowering the pool of assembly-competent fimbrial subunits. In the mechanical framework established above, this reduction in fimbrial density would relieve drag and steric hindrance around the polar flagellum, explaining the enhanced swimming of the *fimC*-overexpression strain despite unchanged transcription of flagellar genes.

The combined biofilm phenotypes of the *fimA*, *fimC*, and *fimD* variants therefore point to a layered adhesion strategy. Under conditions where the CU pathway is intact and *fimA* is upregulated at low temperature, type 1 fimbriae dominate early surface capture and set the geometric and mechanical boundaries of biofilm morphogenesis, producing thick, temperature-robust structures. When key assembly nodes are compromised through loss of the usher, loss of the main chaperone, or misbalanced chaperone levels, D12 appears to fall back on genome-encoded autotransporter adhesins and matrix-associated proteins. In *Pseudomonas aeruginosa*, the matrix adhesin *CdrA* can, together with exopolysaccharides such as Psl, build aggregates that do not depend on the major exopolysaccharide pathways and strengthen the biofilm matrix through multivalent CdrA–CdrA and CdrA–EPS interactions (55). CdrA-like adhesins or related outer-membrane proteins in D12 are plausible candidates for the redundant surface and cell–cell linkages that sustain compact but topologically distinct biofilms in Δ*fimC* and Δ*fimD* backgrounds.

Taken together with the regulatory and mechanical analyses above, these observations are compatible with a model in which the *fimACD* locus of *P. fragi* D12 sits at the junction of local c-di-GMP control, mechanical coupling of the polar flagellum to surfaces, and redundant adhesion modules that safeguard biofilm formation at low temperatures. The genomic linkage of *fimACD* to a predicted Arc-family regulator and an EAL-domain phosphodiesterase, the cold induction of *fimA* and a *tpbB*-like diguanylate cyclase, and precedents from MSHA-type pili and type IV pilus systems where c-di-GMP-binding ATPases tune pilus dynamics are all compatible with a local second-messenger circuit that modulates the balance between motility and attachment at the cell poles. On this background, changes in fimbrial density and architecture redistribute a largely fixed flagellar capacity between long-range swimming and swarm-based surface colonization without altering core flagellar gene expression, while perturbations of *fimD* and *fimC* shift cells onto alternative autotransporter- and matrix-dependent adhesion routes supported by Agn43-like and *CdrA*-like systems.

This organization is well-suited to polar tundra ecosystems, where psychrotrophic inhabitants must repeatedly alternate between dispersal in meltwater films and stable colonization of chilled mineral or organic surfaces. However, the present work isolates the *fimACD* module in simplified laboratory media and does not yet quantify intracellular c-di-GMP, resolve the activity of the downstream phosphodiesterase, or visualize fimbrial dynamics at the single-cell level. Direct measurements of c-di-GMP at 4°C and 15°C, combined with targeted perturbation of the adjacent DGC/PDE pair, and high-resolution structural and single-cell approaches such as cryo-electron microscopy and single-cell omics in simulated soil or sediment matrices will be required to link the proposed local signaling circuit, the mechanical “clutch” behavior of the fimbrial apparatus, and the redundant adhesion network to cold adaptation and ecological fitness *in situ*.

## MATERIALS AND METHODS

### Strains, plasmids, and growth conditions

*P. fragi* strain D12 (culture collection accession: CCTCC No. M2025126) was used in this study. Deletion mutants of *fimA*, *fimC,* and *fimD* were generated by a double-crossover allelic-exchange strategy using plasmid pK18mobsacB (Miaoling Plasmids, China). Overexpression strains were constructed by cloning the target genes into the pUCP18 vector (Miaoling Plasmids, China) and introducing the constructs into wild-type D12 by electroporation, followed by antibiotic selection. Detailed procedures are provided in Text S1 to S7; plasmids and primers are listed in Tables S1 and S2. Unless stated otherwise, all strains were grown in Luria-Bertani (LB) broth (1% [wt/vol] NaCl, 0.5% [wt/vol] yeast extract, and 1% [wt/vol] peptone, pH 7.0), with the addition of kanamycin (100 μg·mL⁻¹) for plasmid-bearing strains (overexpression strains and a wild-type strain carrying the empty pUCP18 vector, WT-EV) to ensure selection. Strains were initially inoculated at a ratio of 1% (vol/vol) and incubated at 4°C, 15°C, or 30°C (180 r min⁻¹) until an OD_600_ ≈ 0.6. These cultures were then used as the standardized inoculum to seed fresh LB medium (1%, vol/vol) for phenotypic assays, where they were further grown to the specific OD_600_ values required for subsequent measurements. Three biological replicates were performed for each condition.

### Transcriptomic analysis and operon structure prediction

Previously obtained raw RNA-seq reads were re-analyzed. Differentially expressed genes (DEGs) were identified using the voom–limma framework (56, 57), which accounts for mean–variance heterogeneity; genes with a false discovery rate (FDR) ≤ 0.05 were considered significant. Functional and pathway enrichment analyses of significant genes were performed using clusterProfiler (58) and gene set enrichment analysis (GSEA) (59) (q < 0.05). The full analysis pipeline is described in Text S7 to S19. The construction of operon maps involved predicting operon boundaries across the *P. fragi* D12 genome using the Operon-mapper web server (https://biocomputo.ibt.unam.mx/operon_mapper/) and subsequently visualizing these genomic vicinities and annotations within SnapGene 8.0 (Dotmatics, USA). For genes with uncharacterized or hypothetical functions, structural modeling and functional inferences were conducted through the AlphaFold Protein Structure Database (pLDDT > 70) to predict biological roles based on high-confidence three-dimensional protein folding.

### Gene expression validation (qRT-PCR)

qRT-PCR was performed using SYBR Green chemistry on a QuantStudio 5 real-time PCR system. Reactions employed SYBR Green Master Mix (Applied Biosystems). 16S rDNA was used as the internal reference gene, and relative expression levels were calculated using the 2^−ΔΔCt^ method (60). Primer sequences are listed in Table S3.

### Phenotypic assays

#### Motility assays

Swimming and swarming motility were assessed on agar plates prepared with distinct agar concentrations and nutrient compositions (61). The swimming medium consisted of 1% (wt/vol) tryptone, 0.5% (wt/vol) NaCl, 0.25% (wt/vol) glucose, and 0.3% (wt/vol) agar, whereas the swarming medium contained 25% (vol/vol) LB broth, 0.5% (wt/vol) glucose, and 0.5% (wt/vol) agar. For the swimming assay, a 0.2 μL aliquot of the bacterial suspension (OD_600_ ≈ 0.2) was inoculated vertically into the center of the semi-solid agar by penetrating the medium with a micropipette tip. To prevent surface interference and ensure accuracy, the exterior of the tip was carefully wiped with sterile filter paper to remove any residual liquid before inoculation. In contrast, swarming motility was determined by spotting a 2 μL aliquot of the suspension directly onto the center of the agar surface. Plates for both swimming and swarming assays were incubated at 4°C, 15°C, and 30°C for 48 h at each temperature. Migration diameters were measured following incubation to quantify the motility performance of each strain.

#### Negative-staining transmission electron microscopy (TEM) of pili

Shaking was stopped, and the cultures were left undisturbed at the same temperature until a visible sediment formed at the bottom of the flasks (62). Bacterial clumps from the sediment were collected with a sterile toothpick, transferred into 2.5% (vol/vol) glutaraldehyde, fixed at room temperature in the dark for 30 min, and stored at 4°C until use. For negative staining, 10–20 μL of the fixed cell suspension was placed on a 200-mesh Formvar-coated copper grid and allowed to adsorb for 10 min. Excess liquid was removed with filter paper, and the grid was air-dried. A 20 μL drop of uranyl acetate solution was applied to the grid for 5 min, excess stain was removed, and the grids were dried under an incandescent lamp. Pili were examined and imaged with a JEM-1400 transmission electron microscope (JEOL, Japan).

#### Biofilm formation assay

Biofilm formation was quantified using a 96-well crystal violet assay (63). Unless stated otherwise, strains were cultivated in LB and standardized as described under “Strains, plasmids, and growth conditions.” Cultures were adjusted to an OD600 ≈ 0.02 in fresh LB, and 200 µL aliquots were transferred into sterile flat-bottom 96-well polystyrene plates. Plates were sealed to prevent evaporation and incubated statically at 4°C, 15°C, or 30°C for 6, 12, 24, and 48 h. After incubation, wells were emptied, washed three times with 250 µL sterile saline to remove nonadherent cells, and then air-dried. Biofilms were stained with 200 µL of 1% (wt/vol) ammonium oxalate–crystal violet solution for 20 min at room temperature, followed by four saline washes. Bound dye was extracted with 200 µL of 95% (vol/vol) ethanol for 20 min under gentle shaking. Absorbance was recorded at 562 nm using a microplate reader (BMG, Germany). Each condition was tested in triplicate, with sterile medium as the blank.

#### Confocal laser scanning microscopy of biofilm three-dimensional structure

Unless stated otherwise, strains were cultivated in LB and standardized as described under “Strains, plasmids, and growth conditions.” Standardized cultures were diluted to 1 × 10^5^ cells per dish in fresh LB and transferred into glass-bottom confocal dishes (35-mm class) in a final volume sufficient to fully cover the glass area (typically 2 mL). Biofilms were allowed to develop statically for 48 h at 4°C, 15°C, or 30°C. For live/dead staining, stock solutions of calcein-AM and propidium iodide (PI) were equilibrated to room temperature for 30 min. An intermediate PI solution was prepared by adding 5 µL of 16 mM PI stock to 10 mL phosphate-buffered saline (PBS; pH 7.4) and vortexing for 1 min. Calcein-AM stock (4 mM, 5 µL) was then added and vortexed for 2 min, giving a mixed working solution containing 2 µM calcein-AM and 8 µM PI. Biofilms were gently washed three times with precooled PBS (pH 7.4) to remove residual esterase activity from the culture medium. The mixed staining solution was added to fully cover the biofilm and incubated for 30 min at room temperature. The staining solution was aspirated immediately before imaging. Biofilms were examined with a Leica TCS SP8 confocal laser scanning microscope (Leica Microsystems, Germany); fluorescence was recorded using excitation/emission settings appropriate for calcein-AM (green) and PI (red), and z-stacks were collected to reconstruct the three-dimensional biofilm architecture (64).

#### Growth curves

To determine the growth characteristics under different temperature regimes, the standardized cultures (OD_600_ ≈ 0.6) were re-inoculated into fresh LB medium at a 1% (vol/vol) ratio and incubated at 4°C, 15°C, and 30°C, respectively. At 4 h intervals, a 200 μL aliquot was aseptically withdrawn from each culture and transferred to a 96-well plate. The optical density at 600 nm (OD_600_) was measured using a microplate reader (BMG, Germany).

### Data analysis and statistics

All experiments were performed at least in triplicate. Data are presented as mean ± standard deviation (mean ± SD). Multiple comparisons were evaluated by one-way analysis of variance (one-way ANOVA), followed by Tukey’s post-hoc test. Statistical analyses were performed using SPSS Statistics version 27 (IBM Corp.). Graphs were prepared in GraphPad Prism v.10.1.2. Transcriptome visualizations were produced in R (v.4.3.2), and final figure assembly was performed in Adobe Illustrator 2023.

## Data Availability

The authors declare that all relevant data supporting the findings of this study are available within the article and its supplemental material or from the corresponding author upon request.

## SUPPLEMENTAL MATERIAL

Supplemental material aem.02472-25-s0001.doc Text S1 to S19, Fig. S1 to S4, and Tables S1 to S3.

ASM does not own the copyrights to Supplemental Material that may be linked to, or accessed through, an article. The authors have granted ASM a non-exclusive, world-wide license to publish the Supplemental Material files. Please contact the corresponding author directly for reuse.
