# Supplementary material for: A cold-responsive fimACD chaperone–usher operon tunes motility and biofilm formation in Pseudomonas fragi D12
Source: Appl Environ Microbiol. 2026 Apr 20;92(5):e02472-25. doi: 10.1128/aem.02472-25 (PMC13188923; doi:10.1128/aem.02472-25)
Supplement: Supplemental material — Text S1 to S19, Fig. S1 to S4, and Tables S1 to S3. [file aem.02472-25-s0001.doc]

**Supplementary Information**

**For**

**A cold-responsive *fimACD* chaperone–usher operon controls motility and biofilm formation in *Pseudomonas fragi* D12**

*Shaoyu Li, ^a, b^ Xia Zhang, ^a, b^ Murong Li, ^a, b^ Meng Zhou, ^a, b^ Zhihao Xing, ^a, b^ Wenbo Zhu, ^d^ Yehui Liu, ^a, b^ Qiyun Li, ^a, *^ Xueli Zang, ^c, *^ Sitong Zhang ^a, b, *^*

^a^The Key Lab of Straw Comprehensive Utilization and Black Soil Protection, Ministry of Education, Jilin Agricultural University, Changchun, 130118, PR China

^b^College of Life Sciences, Jilin Agricultural University, Changchun, 130118, PR China

^c^College of Food and Drug, Changchun Medical College, Changchun, 130031, PR China

^d^Jilin Agricultural Environmental Protection and Rural Energy Management Station, Changchun, 130022, PR China

**CONTENT**

[Text S1. Upstream and downstream homology arm amplification and cross-linking 3](#__RefHeading___Toc209602296)

[Text S2. Construction of knockout vector 3](#__RefHeading___Toc209602297)

[Text S3. Construction of knockout strains 3](#__RefHeading___Toc209602298)

[Text S4 Amplify target gene fragment 4](#__RefHeading___Toc209602299)

[Text S5. Construction of overexpression vector 4](#__RefHeading___Toc209602300)

[Text S6. Construction of overexpression strains 5](#__RefHeading___Toc209602301)

[Text S7. Genetic stability test of recombinant strains 5](#__RefHeading___Toc209602302)

[Text S8: Raw data quality assessment 5](#__RefHeading___Toc209602303)

[Text S9: Read trimming and quality filtering 6](#__RefHeading___Toc209602304)

[Text S10: Reference genome retrieval and index building 6](#__RefHeading___Toc209602305)

[Text S11: Read alignment and post-processing 6](#__RefHeading___Toc209602306)

[Text S12: Gene-level quantification 7](#__RefHeading___Toc209602307)

[Text S13: Filtering and between-sample normalization 7](#__RefHeading___Toc209602308)

[Text S14: Variance modelling and statistical testing (limma-voom) 7](#__RefHeading___Toc209602309)

[Text S15: Preparation of annotation resources 8](#__RefHeading___Toc209602310)

[Text S16: KEGG over-representation analysis 8](#__RefHeading___Toc209602311)

[Text S17: GO enrichment (BP, MF, CC) 8](#__RefHeading___Toc209602312)

[Text S18: COG functional category enrichment 9](#__RefHeading___Toc209602313)

[Text S19: Gene set enrichment analysis (GSEA) 9](#__RefHeading___Toc209602314)

[Figure S1: qRT-PCR validation of differential transcriptomics data 9](#__RefHeading___Toc209602315)

Figure S2: Verification of fim operon transcript levels in *Pseudomonas* *fragi* D12 engineered strains 10

Figure S3: Transcriptional analysis of key flagellar genes in *Pseudomonas* *fragi* D12 fim variants 11

[Figure S4: The principle of dual switching 12](#__RefHeading___Toc209602316)

[Table S1: Plasmid used in the experimen 13](#__RefHeading___Toc209602317)

[Table S2: Primers used in the experimen. 13](#__RefHeading___Toc209602318)

[Table S3: Primers used for qPCR 15](#__RefHeading___Toc209602319)

## Text S1. Upstream and downstream homology arm amplification and cross-linking

Using the whole genome sequence of Pseudomonas fragi D12 as template, we designed specific primers with Primer Premier 5 to amplify the upstream and downstream homology arms. The upstream and downstream arms were then fused by overlap‐extension PCR. Fragments in which the overlap was successful were used as templates to amplify the full‐length construct, which was gel‐purified. The resultant product was stored at −20 °C.

## Text S2. Construction of knockout vector

The amplified upstream and downstream homology arms were then ligated to the full‐length fragment. The resulting insert was digested with two restriction enzymes and ligated into the pK18mobSacB vector. The recombinant vector was transformed into Escherichia coli DH5α competent cells by heat shock. After recovery at 37 °C and 180 r min^-1^ for 45 min, cells were spread on LB agar containing kanamycin. Plates were incubated inverted at 37 °C for 12 h. Positive clones were screened by colony PCR, and the recombinant plasmid thus verified by sequencing was designated pK18mobSacB‐ΔfimX, and stored at −80 °C until further use.

## Text S3. Construction of knockout strains

Gene knockout uses the double exchange method, the principle of which is shown in Figure S2. *Pseudomonas fragi* D12 was used as the recipient strain. The suicide plasmid pK18mobSacB-ΔfimX was introduced into *Escherichia coli* S17-1/λpir by heat-shock transformation to generate the donor strain. Donor and recipient cultures were activated in LB and grown to mid-log phase (OD_600_ = 0.6). Cells were mixed at a recipient:donor ratio of 1:3–1:6 and concentrated by repeated centrifugation (12,000 × g, 1 min). The resulting cell pellet was deposited onto the centre of a sterile filter placed on an LB agar plate without antibiotics and the mating mixture was incubated at 30°C for 24 h. Conjugation mixtures were collected, serially diluted, and plated onto LB agar supplemented with ampicillin (100 μg mL⁻¹) and kanamycin (100 μg mL⁻¹); plates were incubated at 30°C for 24 h to select for the first homologous recombination. Putative integrants were counter-selected on medium containing 15% (w/v) sucrose at 30°C for 48 h, and colonies displaying a kanamycin-sensitive/ampicillin-resistant phenotype were screened as candidates for the second recombination. Deletion of the target locus was verified by PCR with locus-specific primers and confirmed by Sanger sequencing. The confirmed knockout strain was designated *P. fragi* D12-ΔfimX.

## Text S4 Amplify target gene fragment

Using the complete genome of *Pseudomonas fragi* as a template, primers for amplification of the upstream and downstream homology arms were designed with Primer Premier 5 (1). PCR products were checked for specificity by agarose-gel electrophoresis. Gel slices containing the target fragments were purified using the TaKaRa MiniBEST Agarose Gel DNA Extraction Kit according to the manufacturer’s instructions, and purified fragments were carried forward to subsequent procedures.

## Text S5. Construction of overexpression vector

The pUCP18 plasmid was selected as the cloning/overexpression backbone; because pUCP18 carries a β-lactamase (ampicillin) marker and the strain *P. fragi* D12 is ampicillin-resistant, a kanamycin resistance cassette was introduced into pUCP18 to enable reliable selection. The kanamycin (kanR) fragment was amplified from pPIC9K using primers kanR-F and kanR-R (Table S2). PCR products were gel-purified and submitted to Sangon Biotech (Shanghai) for Sanger sequencing; after sequence confirmation, the kanR fragment and pUCP18 were digested with SalI and XbaI and ligated. The ligation products were transformed into chemically competent *Escherichia coli* DH5α by heat shock, and colonies were selected on LB agar supplemented with kanamycin. Positive transformants were verified by colony PCR and Sanger sequencing; the confirmed construct was designated pUCP18-kanR.

Using the pUCP18-kanR backbone, the target gene fimX was PCR-amplified from the *P. fragi* genomic template with gene-specific primers. The agarose-purified fimX amplicon and linearized vector were digested with the appropriate restriction enzymes and ligated using T4 DNA ligase (16°C, 12 h). The ligation mixture was transformed into *E. coli* DH5α by heat shock and kanamycin-resistant colonies (100 μg mL⁻¹) were screened by colony PCR. Positive clones were sequence-verified by Sangon and the correct recombinant plasmid (pUCP18-kanR-fimX) was stored at −80°C for subsequent experiments.

## Text S6. Construction of overexpression strains

Plasmids carrying the overexpression construct were introduced into electrocompetent cells of the parental strain by electroporation (1,800 V, 25 μF, 200 Ω). After recovery, the transformed cultures were spread on LB agar plates supplemented with kanamycin (100 μg·mL⁻¹) and incubated inverted at 30°C for 16–24 h. Putative transformant colonies were screened by colony PCR; PCR-positive cultures were submitted to a commercial sequencing service for confirmation. Clones with the expected sequence were designated *Pseudomonas fragi* D12-OfimX.

## Text S7. Genetic stability test of recombinant strains

Engineered strains were maintained in LB broth at 30°C with shaking at 180 r min^-1^; kanamycin (100 μg·mL⁻¹) was included in the medium for the overexpression strain, and ampicillin (100 μg·mL⁻¹) for the knockout strain. After 20 serial passages, the overexpression strain retained growth on kanamycin-containing plates, whereas the deletion strain showed no *fimX* amplicon by colony PCR.

## Text S8: Raw data quality assessment

Raw sequencing reads (nine FASTQ files total; three biological replicates per temperature condition) were inspected for quality using FastQC (FastQC v0.12.1). Per-sample reports (per-base quality, GC content, sequence duplication levels, over-represented sequences and putative adapter contamination) were generated and then aggregated into a single, study-level HTML summary using MultiQC (2) to facilitate global assessment of data quality and between-sample comparisons.

## Text S9: Read trimming and quality filtering

Adapter removal and quality trimming were performed with Trimmomatic (v0.39) (3). Adapter clipping employed the ILLUMINACLIP setting with up to 2 mismatches, a palindrome clip threshold of 30 and a simple clip threshold of 10. Subsequently, reads were end-trimmed to remove bases with quality scores < 3, a sliding-window trimming (window size = 4 bp) was applied to remove regions with mean quality < 15 (SLIDINGWINDOW:4:15), and only reads ≥ 36 bp after trimming were retained for downstream analyses. These parameters follow commonly used recommendations for Illumina RNA-seq preprocessing.

## Text S10: Reference genome retrieval and index building

The *Pseudomonas fragi* D12 reference genome (RefSeq accessions CP104861–CP104862) was downloaded from NCBI RefSeq and used as the mapping template. A genome index was built using hisat2-build (HISAT2 v2.2.1) prior to read alignment.

## Text S11: Read alignment and post-processing

Quality-filtered reads were aligned to the *P. fragi* D12 reference using HISAT2 (v2.2.1). Although HISAT2 is a splice-aware aligner, bacterial genomes rarely contain spliceosomal introns; the splice-aware capability does not harm bacterial alignments and can be disabled if desired. Alignments were produced in SAM format using 8 CPU threads. SAM files were converted to coordinate-sorted BAM and indexed with SAMtools (v1.17) (4); intermediate SAM files were removed after conversion to conserve disk space.

## Text S12: Gene-level quantification

Gene-level read counts were obtained with featureCounts (subread package; featureCounts v2.0.3) (5) using the sorted BAM files and a GFF3-format annotation. Counting was run in non–strand-specific mode with feature type set to "gene" and attribute "gene_id". featureCounts produced a raw read-count matrix (genes × samples) that served as input for downstream differential-expression analyses.

## Text S13: Filtering and between-sample normalization

Differential-expression analysis was conducted in R (v4.3.2) using the Bioconductor packages edgeR (6) and limma (7). The raw count matrix (after removing non-numeric annotation columns) was read into R, sample columns were labeled to reflect temperature treatments (4°C, 15°C, 30°C) and biological replicates, and an experimental design matrix was constructed specifying temperature as the grouping factor. Low-expression genes were filtered with filterByExpr (edgeR) to retain genes with sufficient counts for reliable testing. A DGEList was created and TMM (8) (trimmed mean of M-values) normalization was applied to account for library-size/compositional differences.

## Text S14: Variance modelling and statistical testing (limma-voom)

We employed the limma–voom pipeline for differential testing. Counts were transformed via voom (9) to obtain log-CPM values and observation-level precision weights that stabilize the mean–variance relationship. Linear models were fitted with lmFit using the design matrix; contrasts for the three pairwise comparisons (15°C vs 4°C, 30°C vs 4°C, 30°C vs 15°C) were specified with make Contrasts and applied with contrasts. fit. Empirical Bayes moderation (eBayes) improved variance estimates across genes. Differential-expression tables were extracted with topTable, and p-values were adjusted for multiple testing using the Benjamini–Hochberg procedure; genes with FDR ≤ 0.05 were considered significant.

## Text S15: Preparation of annotation resources

Because *P. fragi* is not a standard model organism with built-in annotations in many enrichment packages, custom annotation mappings were constructed for KEGG, GO and COG analyses:

**KEGG:** pathway and gene–pathway mappings were retrieved via the KEGG REST API using the Bioconductor KEGGREST package and assembled into a gene-to-pathway mapping table.

**GO:** GO annotations were retrieved from UniProt and/or NCBI Gene and augmented by homology-based transfer where necessary; biomaRt (10) (or equivalent annotation retrieval tools) was used to help map identifiers and retrieve GO terms for gene sets (BP/MF/CC ontologies).

**COG:** COG classifications were obtained from the NCBI COG resource (or via precomputed annotation files); where necessary, BLAST-based assignment was used to map *P. fragi* genes to COG categories.

## Text S16: KEGG over-representation analysis

KEGG pathway enrichment on significant gene lists (per temperature contrast) was performed using enricher (clusterProfiler) (11). Analyses used the custom gene–pathway mapping and applied a hypergeometric test; p- and q-value thresholds were set at 0.05. Results were reported separately for each pairwise contrast.

## Text S17: GO enrichment (BP, MF, CC)

GO-term enrichment was performed independently for Biological Process (BP), Molecular Function (MF) and Cellular Component (CC) ontologies, again using enricher and the custom GO mapping. Biological interpretations emphasized pathways and processes known or plausibly involved in bacterial cold response (metabolic shifts, stress-response systems, transporters and regulatory factors). Statistical thresholds were *p* < 0.05 and q < 0.05.

## Text S18: COG functional category enrichment

COG-category enrichment (using the same enricher framework and COG mappings) highlighted bacterial-specific functional shifts (for example, energy production [C], amino-acid metabolism [E], transcription regulation [K], cell-wall biosynthesis [M], and protein folding/chaperones [O]) across the temperature comparisons.

## Text S19: Gene set enrichment analysis (GSEA)

To capture coordinated but individually modest changes, preranked GSEA (12) was performed on log2 fold-change–ranked gene lists for each contrast. KEGG, GO (BP/MF/CC) and COG gene sets were tested for enrichment using the canonical GSEA permutation framework; multiple-testing adjustment used Benjamini–Hochberg and significance thresholds were set at adjusted p < 0.05. GSEA complements over-representation tests by detecting pathway-level, coordinated patterns even when few individual genes pass the FDR cutoff.

##
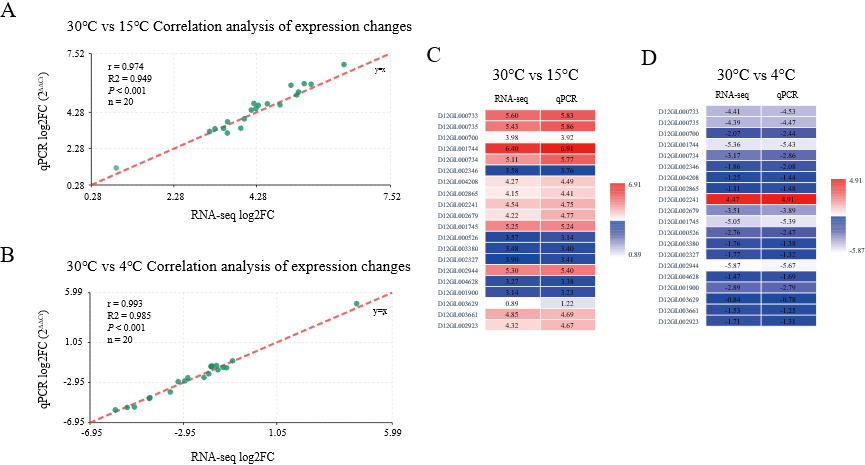
 Figure S1: qRT-PCR validation of differential transcriptomics data

**A, B** qRT-PCR validation of 20 differentially expressed genes was performed at 15°C (A) and 4°C (B). Linear regression analysis showed high consistency between RNA-seq and qRT-PCR data, with R² values of 0.949 and 0.985, respectively. **C, D** Correlation plots of log₂FC values between RNA-seq and qRT-PCR at 15°C (C) and 4°C (D), further verifying the reliability of the transcriptome results.


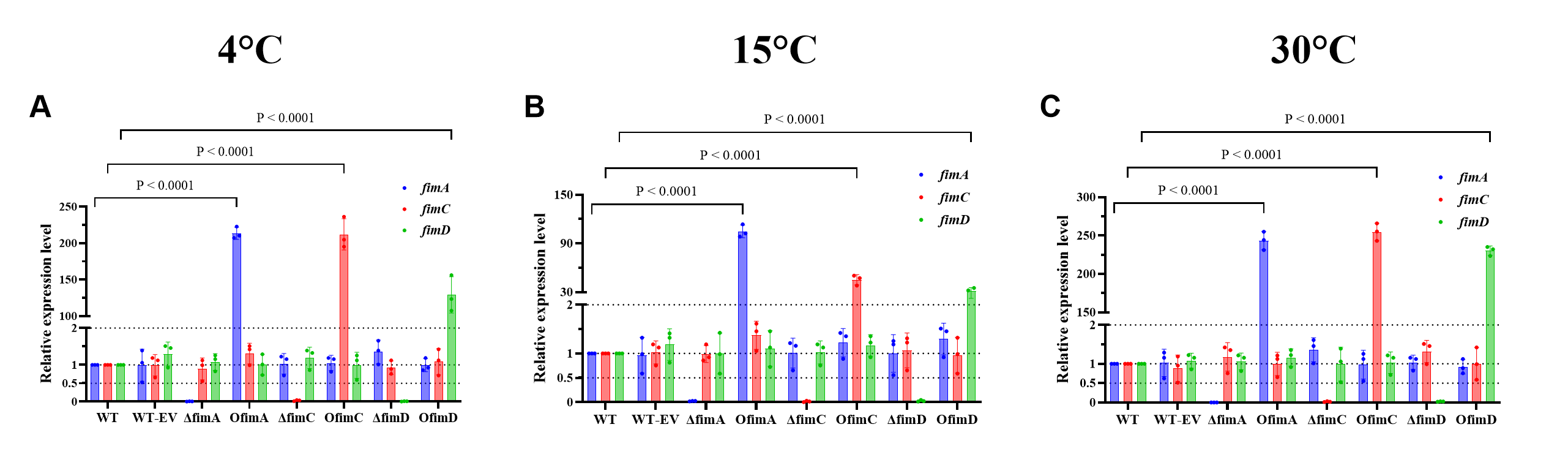


**Figure S2: Verification of *fim* operon transcript levels in *Pseudomonas fragi* D12 engineered strains**

Relative expression levels of *fimA* (blue bars), *fimC* (red bars), and *fimD* (green bars) were determined by qRT-PCR at 4°C **(A)**, 15°C **(B)**, and 30°C **(C).** Strains tested include the wild-type (WT), the empty-vector control (WT-EV), deletion mutants (*ΔfimA*, *ΔfimC* and *ΔfimD*), and overexpression strains (*OfimA*, *OfimC*, and *OfimD*). The 16S rDNA gene was used as the internal reference. Data are presented as the mean ± SD from three biological replicates. Statistical significance was evaluated using two-way ANOVA followed by Dunnett’s multiple comparisons test in GraphPad Prism v.10.1.2. Significant differences relative to the WT are indicated (*p* < 0.0001).


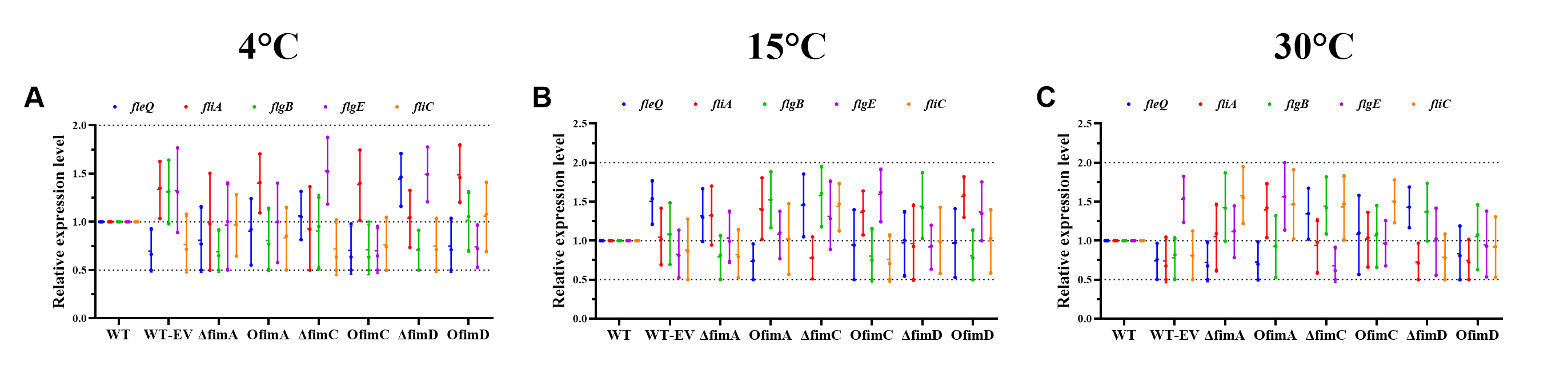


**Figure S3: Transcriptional analysis of key flagellar genes in *Pseudomonas fragi* D12 *fim* variants.**

Relative expression levels of five regulatory and structural flagellar genes *fleQ* (blue), *fliA* (red), *flgB* (green), *flgE* (purple), and *fliC* (orange) were quantified by qRT-PCR at 4°C (A), 15°C (B), and 30°C (C). Strains tested include the wild-type (WT), the empty-vector control (WT-EV), deletion mutants (*ΔfimA*, *ΔfimC* and *ΔfimD*), and overexpression strains (*OfimA*, *OfimC*, and *OfimD*). Data were normalized to the 16S rDNA internal reference and are presented as the mean ± SD from three biological replicates. Gray dashed lines indicate fold-change thresholds (e.g., 0.5, 1.5, and 2.0) relative to the WT. Statistical significance was evaluated using two-way ANOVA followed by Dunnett’s multiple comparisons test. No significant differences in flagellar gene transcript levels were detected between the engineered strains and the WT under any condition tested (*P* > 0.05).


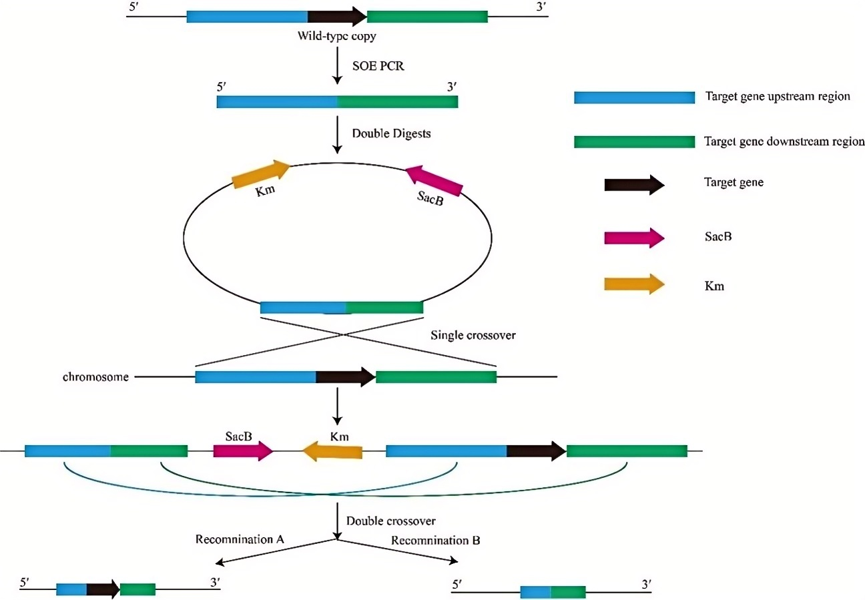


## Figure S4: The principle of dual switching

## Table S1: Plasmid used in the experimen

| Plasmids | Use |
| --- | --- |
| pPIC9K | Provide kanR fragment template |
| pUCP18 | Overexpression plasmid construction for this study |
| pK18mobSacB | Knockout plasmid construction for this experiment |
| pK18mobSacB-ΔfimA | Plasmid used for *fimA* knockout |
| pK18mobSacB-Δ*fimC* | Plasmid used for *fimC* knockout |
| pK18mobSacB-Δ*fimD* | Plasmid used for *fimD* knockout |
| pUCP18-kanR | Overexpression plasmid construction for this experiment |
| pUCP18-kanR-fimA | Plasmid used for *fimA* overexpression |
| pUCP18-kanR-*fimC* | Plasmid used for *fimC* overexpression |
| pUCP18-kanR-fimD | Plasmid used for *fimD* overexpression |

## Table S2: Primers used in the experimen.

| Primer name | primer sequences 5’→3’ |
| --- | --- |
| *fimA*-UP-F | ccggaattctgtccaggaacgccaacc |
| *fimA*-UP-R | gcggggttatcaagcaaataactggcagcac |
| *fimA*-DOWN-F | tatttgcttgataaccccgcaggagaatcac |
| *fimA*-DOWN-R | tgctctagatgttgaactggtccgtggc |
| *fimC*-UP-F | ctagtctagatcgaccatctgcctgacac |
| *fimC*-UP-R | cgactctttcccctgattctcctgcggggttatttac |
| *fimC*-DOWN-F | accccgcaggagaatcaggggaaagagtcgtggtg |
| *fimC*-DOWN-R | acatgcatgccatcagcccgccgtagg |
| *fimD*-UP-F | ctagtctagagattacaccttgcgttttgatgg |
| *fimD*-UP-R | gatgtttatgcgttaaggtgcaccacgactctttcc |
| *fimD*-DOWN-F | agtcgtggtgcaccttaacgcataaacatcgaggactg |
| *fimD*-DOWN-R | acatgcatgcaagggccaactttaggcgtag |
| *fimA*-UP-F | ccggaattctgtccaggaacgccaacc |
| *fimA*-UP-R | gcggggttatcaagcaaataactggcagcac |
| *fimA*-DOWN-F | tatttgcttgataaccccgcaggagaatcac |
| *fimA*-DOWN-R | tgctctagatgttgaactggtccgtggc |
| *fimC*-UP-F | ctagtctagatcgaccatctgcctgacac |
| *fimC*-UP-R | cgactctttcccctgattctcctgcggggttatttac |
| *fimC*-DOWN-F | accccgcaggagaatcaggggaaagagtcgtggtg |
| *fimC*-DOWN-R | acatgcatgccatcagcccgccgtagg |
| *fimD*-UP-F | ctagtctagagattacaccttgcgttttgatgg |
| *fimD*-UP-R | gatgtttatgcgttaaggtgcaccacgactctttcc |
| *fimD*-DOWN-F | agtcgtggtgcaccttaacgcataaacatcgaggactg |
| *fimD*-DOWN-R | acatgcatgcaagggccaactttaggcgtag |
| ccvf-F | cactcattaggcaccccag |
| ccvf-R | cttatcgccattcgcca |
| kanR-F | acgcgtcgactgagggagccacggttgat |
| kanR-R | tgctctagaggttgaggccgttgagcac |
| *OfimA*-F | cgcggatccttctcctgcggggttatttac |
| *OfimA*-R | ccggaattcgtgctgccagttatttgcttg |
| *OfimC*-F | ccggaattcatggccacttttctgagcgc |
| *OfimC*-R | ctagtctagattcccctgcgaagcatgac |
| *OfimD*-F | ctagtctagagtcgtggtgcacctaaaaacag |
| *OfimD*-R | cgagctcgtcagtcctcgatgtttatgcg |

**Note:** The underlined parts indicate the restriction enzyme sites: *fimA*-DOWN-R and kanR-R: Xba I; *fimA*-UP-F and *OfimA*-R: EcoR I; *OfimA*-F: BamH I; kanR-F: Sal I. *fimC*-UP-F, *fimD*-UP-F, *OfimC*-R, and *OfimD*-F: Xba I; *OfimC*-F: EcoR I; *fimC*-DOWN-R, *fimD*-DOWN-R: Sph I; *OfimD*-R: Sac I.

## Table S3: Primers used for qPCR

| Primer name | primer sequences 5’→3’ |
| --- | --- |
| D 12GL000733 F | GACGTTTGTCGCCCTCAATG |
| D 12GL000733 R | GGTGTTGCGGATTTATGCCC |
| D 12GL000735 F | GCCCTGAACTTTTCACGTCC |
| D 12GL000735 R | GTTTCGTAGCACGCCGAATG |
| D 12GL000700 F | TGAAGACCGCGAGCATTTCG |
| D 12GL000700 R | TCGCCTTTGAGAATAGCCAAG |
| D 12GL001744 F | GACCAATCCCAGTCTCCACG |
| D 12GL001744 R | TTCGGTGCGGACTTGAAGAA |
| D 12GL000734 F | GGTGAAAAAGGCGTTGGACG |
| D 12GL000734 R | TGATGCCGAAAAGGTGGGAT |
| D 12GL002346 F | GGTTGCACTGTCTTCGTTGG |
| D 12GL002346 R | ATGGCTTCACCCAAACCCAC |
| D 12GL002865 F | CCCAGAGAAGTCGAAAAGCTG |
| D 12GL002865 R | TGAAACGGCTTCTGGATAGTT |
| D 12GL002241 F | TCAATACCTACACCGCCTGC |
| D 12GL002241 R | ACACCTTGCGTTTTGATGGC |
| D 12GL002679 F | TGTAATCGGTCCCAGCATCG |
| D 12GL002679 R | GGGTTGCCCGTTATCCAGAA |
| D 12GL001745 F | ATCGAGTACGTTCAAGGCCC |
| D 12GL001745 R | CCATCAGAGATGAGGACGGC |
| D 12GL000526 F | AGGTAACTGGGACATCGTGG |
| D 12GL000526 R | AGGCGTTTTTGGGTGTGGAT |
| D 12GL003380 F | CCGGCATTTGGAAAGCAGAG |
| D 12GL003380 R | CGGAGATCACCTTGCCAGTC |
| D 12GL002327 F | CAACTTCGGCCAGCTCAAAA |
| D 12GL002327 R | GCCTGCTGTCAGGTTGATCT |
| D 12GL002944 F | TACCCAGGTGTCACCCTCAA |
| D 12GL002944 R | ACTTAGCTTGGATCGGGCAG |
| D 12GL004628 F | ATCCGAATATCACCGCCACC |
| D 12GL004628 R | ATTGCCCGCTGCCAGAATTA |
| D 12GL001900 F | GGTCAGATCAATGCCTGGGA |
| D 12GL001900 R | CACACACTTCCGTCACGACT |
| D 12GL003692 F | GATCAAGAGCCTTGAGCCAC |
| D 12GL003692 R | TCGCGGAACATCTGAATGGT |
| D 12GL003661 F | GTGAAGACCACGCGCTGAAG |
| D 12GL003661 R | ACACCGTGATACTGGCGTTCT |
| D 12GL002923 F | AAGAACACTGACCCGTCCAC |
| D 12GL002923 R | TCGCTTGTGGGAACATCTCC |
| D 12GL001900 R | CACACACTTCCGTCACGACT |
| D 12GL003692 F | GATCAAGAGCCTTGAGCCAC |
| D 12GL003692 R | TCGCGGAACATCTGAATGGT |
| D 12GL003661 F | GTGAAGACCACGCGCTGAAG |
| D 12GL003661 R | ACACCGTGATACTGGCGTTCT |
| D 12GL002923 F | AAGAACACTGACCCGTCCAC |
| D 12GL002923 R | TCGCTTGTGGGAACATCTCC |
| *fimA* F | TACCCAACCACTGCATTCCC |
| *fimA* R | TGCCATCAAAACGCAAGGTG |
| *fimC* F | ATACGGGCGATGCAAGTCAA |
| *fimC* R | TCTTGTGCCAGTCCTTTCCC |
| *fimD* F | CAAACAAGGTCAGAGCGTGC |
| *fimD* R | AACCGGAGGTCGAATAACGG |
| *fleQ* F | CATTTCGAGGCTGGATAACACC |
| *fleQ* R | AGGTTTACTTAAGACACTGGCTG |
| *flgB* F | AGCCCTTTGAATTTGCTGTTG |
| *flgB* R | AGAACACAGTGGACGCTC |
| *flgE* F | TCATCGTCAATCACCCGCTC |
| *flgE* R | TCAGGCCCACATCCCGTTC |
| *fliA* F | AATCAAGTCATCCACCTGCAC |
| *fliA* R | CGCATTGCCTATCACTTGCT |
| *fliC* F | TCAGATGTGTCGCCCTTC |
| *fliC* R | GATATAAAGCTAGCCAACACAACC |

**References:**

1. Lalitha S. 2000. Primer premier 5. Biotech Software & Internet Report 1:270–272.

2. Ewels P, Magnusson M, Lundin S, Käller M. 2016. MultiQC: summarize analysis results for multiple tools and samples in a single report. Bioinformatics 32:3047–3048.

3. Bolger AM, Lohse M, Usadel B. 2014. Trimmomatic: a flexible trimmer for Illumina sequence data. Bioinformatics 30:2114–2120.

4. Li H, Handsaker B, Wysoker A, Fennell T, Ruan J, Homer N, Marth G, Abecasis G, Durbin R. 2009. The Sequence Alignment/Map format and SAMtools. Bioinformatics 25:2078–2079.

5. Liao Y, Smyth GK, Shi W. 2014. featureCounts: an efficient general-purpose program for assigning sequence reads to genomic features. Bioinformatics 30:923–930.

6. Robinson MD, McCarthy DJ, Smyth GK. 2010. edgeR: a Bioconductor package for differential expression analysis of digital gene expression data. Bioinformatics 26:139–140.

7. Ritchie ME, Phipson B, Wu D, Hu Y, Law CW, Shi W, Smyth GK. 2015. limma powers differential expression analyses for RNA-sequencing and microarray studies. Nucleic Acids Res 43: e47.

8. Robinson MD, Oshlack A. 2010. A scaling normalization method for differential expression analysis of RNA-seq data. Genome Biology 11: R25.

9. Law CW, Chen Y, Shi W, Smyth GK. 2014. voom: precision weights unlock linear model analysis tools for RNA-seq read counts. Genome Biology 15: R29.

10. Durinck S, Spellman PT, Birney E, Huber W. 2009. Mapping identifiers for the integration of genomic datasets with the R/Bioconductor package biomaRt. Nat Protoc 4:1184–1191.

11. Wu T, Hu E, Xu S, Chen M, Guo P, Dai Z, Feng T, Zhou L, Tang W, Zhan L, Fu X, Liu S, Bo X, Yu G. 2021. clusterProfiler 4.0: A universal enrichment tool for interpreting omics data. Innovation (Camb) 2:100141.

12. Subramanian A, Tamayo P, Mootha VK, Mukherjee S, Ebert BL, Gillette MA, Paulovich A, Pomeroy SL, Golub TR, Lander ES, Mesirov JP. 2005. Gene set enrichment analysis: a knowledge-based approach for interpreting genome-wide expression profiles. Proc Natl Acad Sci U S A 102:15545–15550.
